# Can Native Vegetation in Agroecosystems Provide a Net Benefit for Pollinators, Despite Pesticide Use?

**DOI:** 10.1093/biosci/biaf194

**Published:** 2026-01-06

**Authors:** Amy L Toth, Adam G Dolezal, Ashley L St. Clair, Edward M Hsieh, Maura J Hall, Kate E Borchardt, Matthew D Stephenson, Steven P Bradbury, John C Tyndall, Matthew E O’Neal, Lisa A Schulte

**Affiliations:** Department of Ecology, Evolution, and Organismal Biology, Iowa State University, Ames, Iowa, 50011, USA; Department of Entomology, University of Illinois, Urbana-Champaign, Urbana, Illinois, 61801, USA; Department of Entomology, University of Illinois, Urbana-Champaign, Urbana, Illinois, 61801, USA; Department of Entomology, University of Illinois, Urbana-Champaign, Urbana, Illinois, 61801, USA; Department of Plant Pathology, Entomology, and Microbiology, Iowa State University, Ames, Iowa, 50011, USA; Department of Ecology, Evolution, and Organismal Biology, Iowa State University, Ames, Iowa, 50011, USA; Department of Biology, University of Massachusetts, Amherst, Massachusetts, 01003, USA; Bioeconomy Institute, Iowa State University, Ames, Iowa, 50011, USA; Department of Plant Pathology, Entomology, and Microbiology, Iowa State University, Ames, Iowa, 50011, USA; Department of Natural Resource Ecology and Management, Iowa State University, Ames, Iowa, 50011, USA; Department of Natural Resource Ecology and Management, Iowa State University, Ames, Iowa, 50011, USA; Department of Plant Pathology, Entomology, and Microbiology, Iowa State University, Ames, Iowa, 50011, USA; Bioeconomy Institute, Iowa State University, Ames, Iowa, 50011, USA; Department of Natural Resource Ecology and Management, Iowa State University, Ames, Iowa, 50011, USA

**Keywords:** conservation, agroecosystems, plant–animal interactions, toxicology, entomology

## Abstract

Pollinator health and population declines are of global concern, with ramifications for biodiversity conservation and the human food supply. Although the establishment of native flowering vegetation is a broadly supported pollinator conservation strategy, concerns about enhancing habitat in agroecosystems with widespread pesticide use remain. To evaluate this trade-off, we synthesized research findings for prairie strips, a model conservation practice that involves patches of diverse, native plants within row-crop fields. Prairie strips enhance pollinator forage quantity and quality, support a more diverse and abundant wild bee community, sustain monarch butterflies, and increase managed honey bee productivity. Although exposure to pesticides may occur, concentrations are typically below levels that have negative impacts on pollinator health. We discuss knowledge gaps and conclude native vegetation enhancements in agricultural landscapes, including those with widespread pesticide use, are a promising strategy for pollinator conservation, with the potential for net positive effects despite pesticide exposure.

Populations of one-quarter of the world’s wild pollinator species are shrinking, with critical implications for crop pollination and sustaining overall biodiversity (Potts et al. 2010). In addition, health issues plague managed pollinators such as honey bees, which humans rely on for food production (Steinhauer et al. 2018). Although there are many causes of pollinator decline, the loss or degradation of habitat providing nesting and floral resources is widely understood to be a major contributor (Potts et al. 2010, Quinlan and Grozinger 2023).

Habitat enhancement through the establishment of native flowering vegetation, even in small patches, is a conservation strategy used in many environments, including crop fields, parks, yards, and along roadsides (Gill et al. 2016). Such enhancement is especially important in agricultural landscapes, which occupy an increasing area of land globally (Garibaldi et al. 2021a). It is crucial to find ways to balance the habitat needs of pollinators, and biodiversity more broadly, with the needs of people (Foley et al. 2005, Lanz et al. 2018).

Although native vegetation enhancements for pollinators are generally viewed as ecologically beneficial (Haaland et al. 2011), questions remain about the potential creation of ecological traps (Ganser et al. 2019). An ecological trap in the context of habitat selection is defined as an area that provides attractive resources to a target species such as food, shelter, or reproductive opportunities but that also contains harmful factors that result in an overall net fitness loss for the species (Hale and Swearer 2016).

The concerns about the potential for ecological traps for pollinators in agroecosystems are numerous because of factors such as soil tillage (Ganser et al. 2019), simplified floral networks (Carman and Jenkins 2016), and pesticide use (Mogren and Lundgren 2016, Raine and Rundlöf 2024). In areas where pesticide use is common, pollinators can be exposed through multiple routes, even if pesticides are not directly applied to their floral resources. Contaminated dust associated with planting pesticide-treated seeds or drift from foliar pesticide applications can move downwind from crops onto nearby flowers (Simon-Delso et al. 2017). Systemic insecticides that move with water can be taken up into noncrop flowers (Mogren and Lundgren 2016). In addition to the potential for acute mortality of pollinators, sublethal exposure to various agrochemicals has been associated with deficits in learning (Siviter et al. 2018), foraging (Stanley et al. 2016), resilience to pathogens (Harwood and Dolezal 2020), thermal physiology (Potts et al. 2018), and reproduction (Wu-Smart and Spivak 2016). Therefore, although habitat enhancement creates floral resources for pollinators, there is concern it could also attract pollinators to forage on contaminated plants, resulting in toxicity and overall negative fitness impacts. Although pesticide exposure can and does occur in natural and seminatural cover located adjacent to conventional agriculture, few studies have considered both the net effects of habitat enhancement and pesticide exposure on measures of pollinator health and populations such as species richness, abundance, individual or nest size, survival (Grant et al. 2021, St. Clair et al. 2025).

Whether the net effect is positive, neutral, or negative has broad implications for environmental policies, pollinator habitat installations, and pest management practices within agricultural lands. Integrating native plantings into agricultural landscapes has the potential to vastly increase wildlife habitat, however, sufficient data are typically lacking to assess both positive and negative effects of forage resources, including nutritional quality and pesticide exposure on a diversity of pollinator species. In the present article, we present a synthesis of a data-rich model system that allows for an informed assessment of both benefits and costs of conservation habitat creation on pollinators.

## Prairie strips: A model system

The purpose of this article is to provide a detailed examination of whether benefits of native plantings have the potential to outweigh the costs of agricultural practices to pollinators, using the model system of prairie strips within conventionally managed corn–soybean fields in the Upper Midwestern United States (box 1, figure 1). After more than a decade of research, prairie strips became a federally supported practice in the United States in 2018. The specific practice, designated under the US Conservation Reserve Program as Conservation Practice-43 Prairie Strips (USDA Farm Service Agency 2019), features a diverse mix of native perennial grass and forb species, established within or at the edge of row-crop fields or in irrigation pivot areas (for details, see box 1). This practice is implemented for agronomic and ecological benefits, primarily reduced soil erosion and nutrient runoff from crop fields for enhanced water quality, but also for carbon sequestration and habitat (Schulte et al. 2017, Kemmerling et al. 2022). Prairie strips are most often implemented within the context of corn and soybean fields, neither of which require insect pollination. Although pollinator conservation is not the primary goal of CP-43 (compared with other, more widespread practices such as CP-42, Pollinator Habitat), this system provides the opportunity to specifically address cost–benefit trade-offs to pollinators for a relatively new practice being increasingly adopted by farmers (Giese et al. 2024). Recently, CP-43 prairie strips were also recognized by the US Environmental Protection Agency (USEPA) as a risk mitigation measure for their potential to impede pesticide spray drift exposure to sensitive species or habitats (USEPA 2022). However, because these habitats can be attractive to pollinators and other beneficial species, they could inadvertently increase their exposure to pesticides as they are often established within treated fields. In the present article, we synthesize findings from studies on prairie strips (box 1) to ask whether this conservation practice in a landscape with widespread pesticide use (box 2) can provide net benefits to pollinators. In a world increasingly dominated by extensive land use for row-crop agriculture, this system is a useful example of an extreme environment in which extensive crop monocultures (figure 2), conventionally farmed using synthetic fertilizers and pesticides (box 3), leaves little room for biodiversity conservation. Our synthesis is based on research addressing the nutritional benefits of native vegetation enhancements and the risks of pesticide exposure for pollinators in the Corn Belt of the upper Midwestern United States.

**Figure 1.**
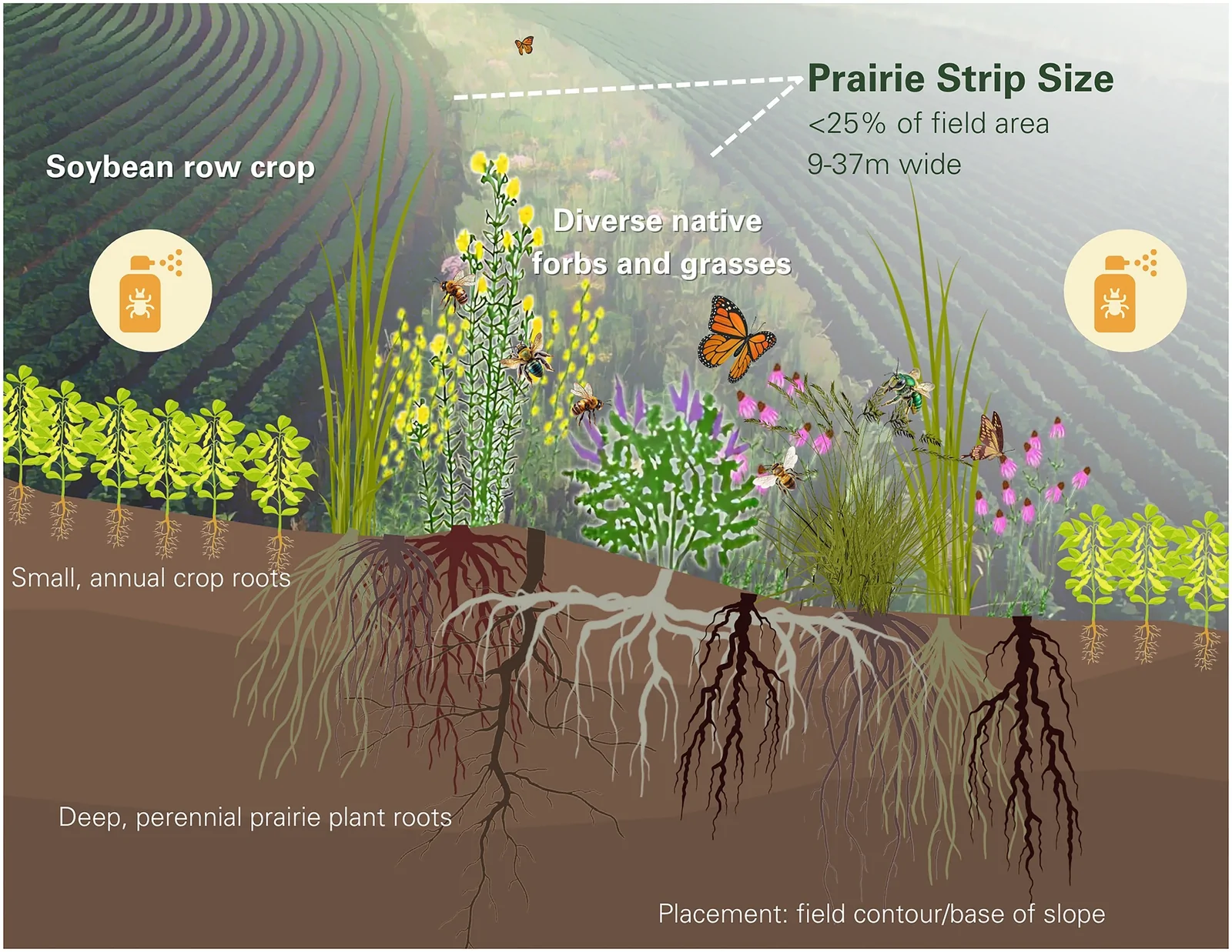
Anatomy of a prairie strip. The layout and structure of a prairie strip within a landscape scale, illustrating below- and aboveground features. The bubble within the soybean field represents pesticide use in crops, including the use of insecticides, fungicides, and herbicides.

Box 1. **What are prairie strips?**

Prairie strips are one of 25 US federal Conservation Reserve Program practices that feature pollinator habitat. Prairie strips can also be implemented through the USDA Environmental Quality Incentives Program, other federal programs, and state or nonprofit programs. Landowners can also implement the practice without support. The definition of prairie strips within agricultural contexts was standardized by USDA in conservation practice code CP-43 in December 2019 (USDA Farm Service Agency 2019). A prairie strip is a linear practice, 9–37 meters in width, composed of diverse, perennial native grasses and forbs (figure 1). They are typically installed in a single year through sowing of a locally or regionally sourced seed mix containing over 30 plant species with a high proportion of forbs, become established over the course of 2–3 years, and can be used long term for multiple years with minimal additional maintenance. Prairie strips can be placed on field contours, terrace channels, next to pivot corners, or at the base of slopes to intercept runoff from crop fields. The specific location of prairie strips within or around a field is determined by field-scale hydrologic conditions, such as water runoff patterns, and crop management operations. Prairie strips can make up up to 25% of a field’s area (recommended more than 10%) and are often installed in areas with marginal crop production. Nearly 10,700 hectares of CP-43 have been implemented across 13 US states since December 2019 (USDA Farm Service Agency 2025). According to the USDA Farm Service Agency, prairie strips are used to “mitigate water erosion, provide habitat for wildlife species, improve water quality by intercepting sediment and nutrients, and sequester carbon” (USDA Farm Service Agency 2019). They are often used in combination with other in and edge-of-field best management practices such as nutrient management, conservation tillage, cover crops, terraces, riparian buffers, saturated buffers, and grass waterways. Studies have shown that prairie strips are effective at improving soil, water, and air quality and habitat for biodiversity (Schulte et al. 2017, Kordbacheh et al. 2020, Stephenson 2022, Dutter et al. 2024, Giese et al. 2024, Stepheson et al. 2025). Prairie strips are among the least costly and most cost-effective water quality conservation practices available to farmers when field conditions are suitable for the practice (Schulte et al. 2017).

Box 2. **Major pesticides used in the US Corn Belt.**

The USDA-sponsored prairie strip practice does not restrict farmers from using pesticides on adjacent crops. With conventionally grown crops, pollinators could be exposed to pesticides, including several broad-spectrum insecticides used at key times of the growing season. Neonicotinoids, specifically clothianidin, thiamethoxam and imidacloprid, are commonly applied to corn and soybean seeds in the north central United States, especially Iowa (Douglas and Tooker 2015). Pyrethroids and organophosphates are the most commonly used foliar insecticides in the corn–soybean production systems; for example, more than 190, 000  kilograms were applied in Iowa by the most recent official estimate in 2018 (US Department of Agriculture 2019). The use of these active ingredients increased soon after the arrival and establishment of the soybean aphid in the Midwest (Yang and Suh 2015). Pyrethroids are commonly used for the soybean aphid and are typically sprayed when soybeans are blooming (Hodgson et al. 2012). In the corn–soybean production region where prairie strips originated, there has been a recent increase in the prolific application of foliar applied fungicides on corn and soybeans (Bandara et al. 2020). The impact of insecticides on bees may be enhanced by fungicides that are applied concurrently (Schuhmann et al. 2022). Applying fungicides is often coupled with insecticides in a tank mix and timed to prevent outbreaks from fungal pathogens. Although many of these pesticides have been documented in prairie strips, the frequency varies with the active ingredient and time of year; concentrations are generally below levels of concern for monarch butterflies and honey bees (Hall 2021, Hall et al. 2022).

Box 3. **Landscape context of prairie strips.**

The Midwestern United States is home of the Corn Belt, an extensive area (figure 2a) where the majority land use (USDA National Agricultural Statistics Service 2024) is row-crop corn and soybean production, often grown using synthetic fertilizers and pesticides. Iowa, where most of the research reviewed in this article was conducted, is the only US state fully encompassed in the Corn Belt and has 72% of its land cover devoted to corn and soybean production (Green et al. 2018). Historically, the state was largely (more than 80%) covered by tallgrass prairie, but less than 0.01% (8100 hectares) of the remnant prairie remains in Iowa, now distributed in very small patches (Smith 1998). Prairie strips, although they are generally small in size, currently cover 2600 hectares within Iowa (USDA Farm Service Agency 2025), contributing in an important way to prairie habitat in the state. Similar to state- and region-wide statistics (USDA National Agricultural Statistics Service 2024), the landcover surrounding farms with prairie strips is dominated by corn and soybean production (figure 2b, 2c).

**Figure 2.**
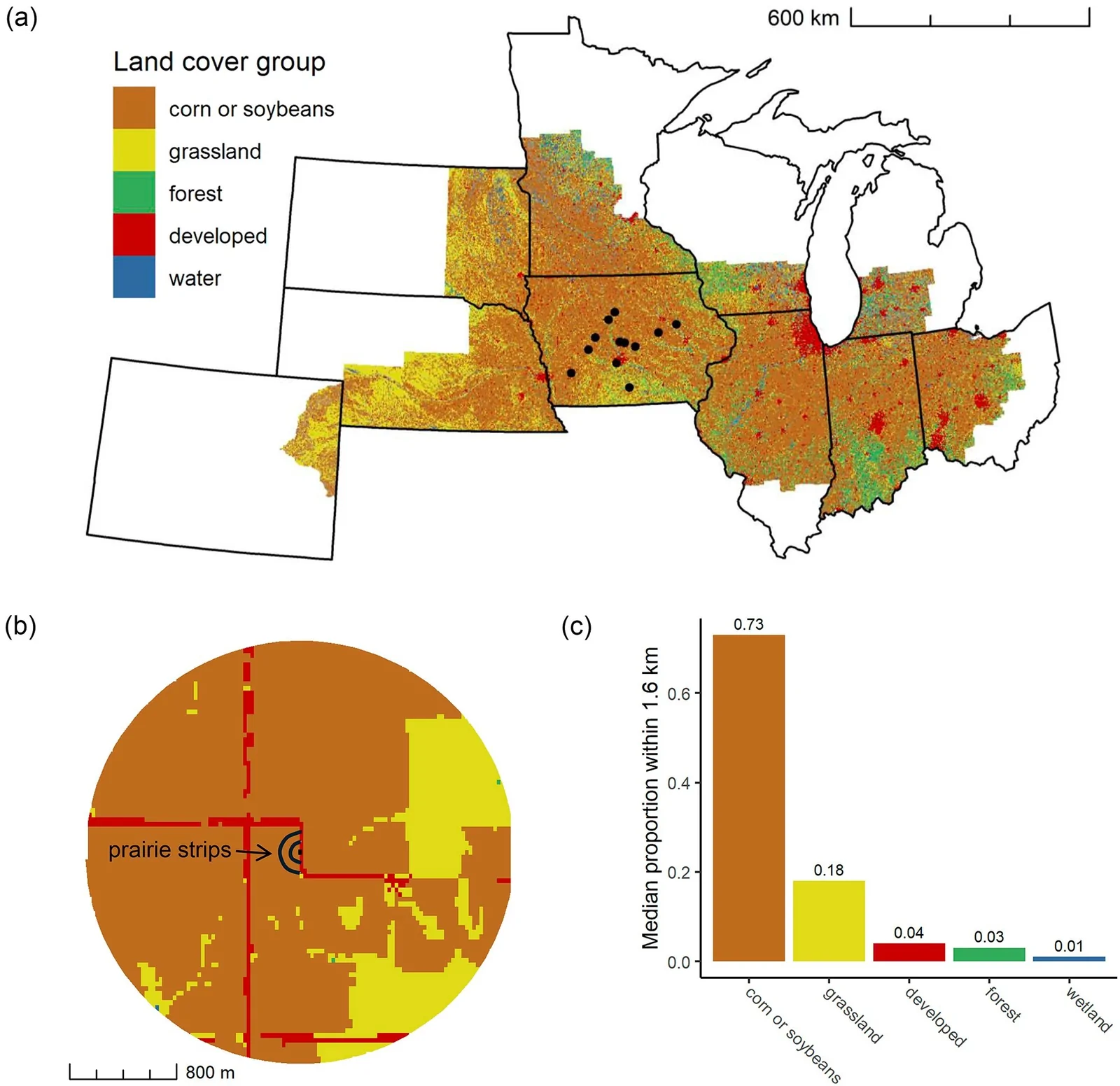
Landscape context of prairie strips. (a) The Corn Belt (the colored region) is an area of extensive corn and soybean production in the upper Midwestern United States, with Iowa at its epicenter. The colors in all of the subfigures represent land cover classes derived from the USDA Cropland Data Layer (USDA National Agricultural Statistics Service 2024). The grassland category includes pastures, hay fields, grassed waterways, buffer or contour strips, and ditches, with only a very small percentage of grassland in Iowa representing restored or remnant prairie habitat. Black dots indicate locations of 12 farm fields with prairie strips in Iowa that were used in prairie strips studies cited in the present article. (b) Land cover within 1.6 kilometers of a field with prairie strips in southwest Iowa, illustrating the location and size of the prairie strips (in black). This site had median landcover values among the sites studied, with a matrix heavily dominated by corn and soybean agriculture (73%), with 21% categorized as grassland in the form of pasture or hay fields and 4% as developed roadways, with 2% other land cover types (water, forest). (c) Median land cover proportions within 1.6 kilometers of the 12 farm fields with prairie strips (the locations are indicated by black dots in panel (a)).

Previous studies in Iowa suggest landscape context is an important factor in pollinator abundance and health (St. Clair et al. 2022b). Although landscape effects related to prairie strips’ value to pollinators have not yet been assessed, the amount of land in corn and soybean production is related to lower wild bee abundance and diversity at soybean farms without prairie strips (St. Clair et al. 2022b). Apiaries in locations with greater amounts of corn and soybean production produce worker honey bees with lower preoverwintering fat stores, a measure of colony health (Dolezal et al. 2016). Although apiaries at locations with high corn–soybean cover actually gain more weight in summer than those with less corn–soybean cover, the loss of floral resources after soybeans cease flowering produce a feast–famine dynamic leading to small, weak colonies prior to overwintering (Dolezal et al. 2019). These findings, along with other studies in this region (Koh et al. 2016, Otto et al. 2016) suggest the Corn Belt landscape is not fully supportive of pollinator health. In general, landscapes devoted to agricultural production provide pollinators with limited habitat options (Garibaldi et al. 2021a). With extremely limited public and private land containing native flowering forbs to sustain pollinators in Iowa (figure 2), prairie strips represent one of the few habitats available.

There are multiple reasons why prairie strips provide a premier model to address the costs and benefits of farm-based pollinator habitat. First, there are a large number of agronomic, ecological, and social publications, with rich data sets, for this model system (for a review, see Schulte et al. 2017, Kemmerling et al. 2022). Second, the Midwest currently has the highest rate of prairie strips adoption and the practice is spreading to additional regions (USDA Farm Service Agency 2025). Third, the Midwest is crucial for wild pollinator conservation (Grixti et al. 2009, Koh et al. 2016) and reversing extremely high honey bee losses (Bruckner et al. 2023). This area is or was home to federally protected pollinator species (Schlicht and Orwig 1998, Wolf et al. 2022), as well as to the monarch butterfly, which is proposed by the US Fish and Wildlife Service as a threatened species under the Endangered Species Act (FWS 2024). Fourth, the data from this region represent a real-world example of conventional agricultural practices with extensive use of multiple pesticides occurring alongside conservation (USDA National Agricultural Statistics Service 2024). The Midwest region is dominated by monocultures of corn and soybean, and as a result, pollinators have limited resources outside of small patches of natural or restored habitat (Gallant et al. 2011). Finally, prairie strips are primarily promoted for their soil health and water quality benefits (box 1); therefore, it is already known that the practice can have broad net benefits for farmers and the environment. Here, we focus on whether there are net benefits, specifically, for pollinators.

In this article, we first describe what is known about the benefits of prairie strips to pollinators, including potential forage, nesting, and reproduction benefits, and then examine potential costs associated with pesticide exposure. We then synthesize these costs and benefits, first considering pollinator specifically, then in a broader societal context, considering stakeholders and the likelihood of implementing sustainable environmental practices in agroecosystems. We end by providing insights from this system that more broadly address pollinator conservation.

## Benefits of prairie strips to pollinators

### Wild pollinators

The goal of practices such as prairie strips is to yield agricultural and conservation benefits for the environment and society. Plant–pollinator mutualisms are highly integrated within ecosystems, from soil microbes to higher trophic levels; therefore, it is essential to consider plant–pollinator interactions within agroecosystems as a whole (Borchardt et al. 2021). Prairie strips offer pollinators a more rich and abundant foraging environment than crop field edges, with three times higher flowering forb richness and six times higher floral abundance (Stephenson et al. 2025). Within crop fields that incorporate prairie strips, greater floral diversity and coverage supported more abundant and species-rich native bee communities (Kordbacheh et al. 2020). Observations of pollinator visitation have pinpointed several key native plants in prairie strips that are used as forage by a large subset of the bee community (Kordbacheh et al. 2020). These results support research in other systems that landscape enhancements in field edges can support bee communities (Jha and Kremen 2013, Morandin and Kremen 2013). In addition to improved floral resources, prairie strips can provide nesting habitat for stem and ground nesting bees, as well as a refuge from tillage, which can disrupt ground nesting species (Williams et al. 2010). Prairie strips were also associated with higher activity densities of adult monarch butterflies (Stephenson et al. 2025) and higher abundance or activity density and diversity of butterflies (Kemmerling et al. 2022), aphid predators (Cox et al. 2014), and ground beetles (i.e., carabids; Vargas 2024).

Networks of plant and pollinator interactions can be used to assess ecological resilience and health of the community (Soares et al. 2017). In prairie strips, there are larger, more complex plant–bee networks than in the field margins of corn–soybean fields without strips (Borchardt et al. 2023). These networks include more interacting bee partners per plant species (i.e., increased likelihood of bee-mediated pollination) and a more nested network structure (Borchardt et al. 2023), which is characteristic of the presence of more rare, specialized species (Bascompte et al. 2003). These improved plant–bee interactions may also positively affect aspects of wild bee health for some species. Large bees found in prairie strips, such as bumble bees, showed evidence of lower fluctuating asymmetry in their wings, suggesting lower developmental stress (Borchardt et al. 2023). In addition, lower levels of wing wear were found for some halictid (i.e., sweat) bee genera in prairie strips than in unenhanced field margins (Borchardt et al. 2023), suggesting less energy spent on foraging. Effects varied by bee taxon, suggesting that not all bee species are responding in the same way to prairie strips.

### Managed honey bees

The lack of diverse, nutritious, and abundant forage is a primary concern for maintaining honey bee health, with ramifications for the sustainability and economics of beekeeping (Potts et al. 2010). Evidence accumulated over the past several decades indicates that landscape simplification, primarily for large-scale farming, is associated with a loss of floral resources and decreased honey production (Smart et al. 2016, Smith et al. 2021).

The Corn Belt of the Upper Midwestern United States represents an extreme example of landscape simplification—for example, more than 70% of land cover is in monocultural crop production in the state of Iowa (Green et al. 2018). This region also experienced some of the highest reported honey bee colony losses in recent years (Bruckner et al. 2023). Prior work suggests that forage availability late in the growing season is critical to honey bee health (Dolezal et al. 2019). Strikingly, colonies transported from crop fields to restored prairie areas at the end of the growing season were rescued from late-season crashes in colony weight and worker bee population (Dolezal et al. 2019). However, these nutritional benefits were only realized if the bees spent spring and early summer in an environment in which there was no or limited exposure to foliar applications of lambda-cyhalothrin, a pyrethroid insecticide (St. Clair et al. 2025). These studies highlight the potential of native perennial vegetation, combined with reduced or careful use of insecticides, to alleviate the challenges honey bees face in areas primarily consisting of row-crop monocultures.

Given the midseason forage boom and the late-season bust in this landscape, prairie strips integrated into corn–soybean fields present an opportunity for bees to benefit from both agricultural crop field and natural prairie derived forage. Indeed, Zhang and colleagues (2023) found that honey bee colonies located in prairie strips were more successful in food collection (i.e., nectar and pollen), population growth, and winter survival than those at farms without prairie strips. This benefit is likely derived from access to nearby abundant and diverse floral resources in strips; 50% of the plant species in the strips were found in pollen collected by honey bees (Zhang et al. 2023). Honey bees apparently switch from foraging on soybean and weedy species in summer to native plant species late in the growing season (Zhang et al. 2021), the previously identified period of forage dearth in this landscape.

These studies suggest that prairie strips may improve honey bee health primarily via increases in food availability, which can positively influence nutritional health and, therefore, survival. The increased food availability could be beneficial to beekeepers in numerous ways—improving queen survival and health (St. Clair et al. 2022a), reducing costly and time-consuming supplemental feeding, and decreasing the costs of replacing lost bees. Additionally, accumulating evidence indicates that access to abundant, diverse, and nutritious forage (especially pollen) can make bees more resilient to other forms of stress, such as pathogens and pesticides (Harwood and Dolezal 2020, Quinlan and Grozinger 2023). Therefore, the improved forage provided by prairie strips has the potential to contribute to overall healthier and more stress-resilient honey bees.

Importantly, honey bees in prairie strips experienced these benefits even though they were concurrently exposed to pesticides used in adjacent fields (detailed information on common compounds and their concentrations can be found below). Although honey bee colonies lived in environments where multiple pesticides are regularly used (e.g., neonicotinoid seed treatments, foliar applications of insecticides and fungicides), colony-level health metrics were improved on fields with prairie strips, indicating an overall net benefit. The positive response of honey bees to prairie strips was even greater than what was observed when honey bees were moved from insecticide-treated soybean fields to field-scale (32–55 hectares) prairie reconstructions (St. Clair et al. 2025). This may be partially explained by the fact that the apiaries that stayed in the prairie strips did not experience the stress of relocation. Also, prairie strips embedded in crop fields may hit a sweet spot by providing honey bees with continuous access to forage from cultivated crops (e.g., soybean), field edge weeds (e.g., clover), and diverse prairie forbs, creating a highly favorable foraging environment during the majority of their active field season. In addition, because they are not treated with insecticides, prairie strips provide a safer area for bees and other insects during direct insecticide application in surrounding fields, essentially acting as buffer zones (Haddaway et al. 2018). Although there are still important questions about the impacts of honey bees on wild bee populations (box 4), the results to date suggest that foraging resources provided by prairie strips are a promising strategy to enhance both managed and wild bee health in monocultural corn–soybean agroecosystems.

Box 4. **Honey bee and wild bee interactions in prairie strips.**

Biodiversity conservation goals can be compromised when commercial beekeeping occurs on conservation lands. Honey bees, a nonnative species in the United States, may compete with wild pollinators for forage and transmit pathogens and pests to native species (Mallinger et al. 2017). Prior studies in other areas of the world have shown honey bees can disrupt plant–pollinator interactions (Valido et al. 2019). Thus far, multiple studies in landscapes dominated by row-crop agriculture such as Iowa, in the United States, have shown no effect of honey bee apiaries on wild bee abundance and diversity in prairies, crop fields, and crop fields with prairie strips. Within reconstructed and remnant prairies in Iowa, wild bee abundance and diversity were the same or higher in prairies that had apiaries present than in those that did not (Pritchard et al. 2021). There was no effect of apiary presence on the abundance, diversity, and community composition of wild bees in soybean fields (St. Clair et al. 2022a). In prairie strips, a large and diverse community of wild bees can exist in prairie strips even in the presence of honey bees from early to midsummer (Borchardt et al. 2023). Using both plant–pollinator networks and bee body condition indicators, there was no effect of the presence of honey bees on wild bee abundance, diversity, and plant–bee interaction network structures (Borchardt et al. 2025). The mechanisms associated with these findings are not yet completely clear, but we expect that the additional and more diverse floral resources introduced to crop fields through prairie strips offset the impact of competition with honey bees. Furthermore, many plant species that are highly favored by some wild bee species are not used by honey bees, which tend to prefer leguminous species such as soybean, clover, and partridge pea (Zhang et al. 2021). However, in restored prairies in this region (not specifically strips), bumble bees at sites with honey bee apiaries had higher titers of some common honey bee viruses (Pritchard et al. 2021), suggesting that there could be some potential for disease spillover from honey bees in prairie strips. Further research should address whether there are effects of honey bees on specific taxa or under certain conditions (e.g., seasons, surrounding landscapes).

## Costs of prairie strips to pollinators

### Pesticide use and exposure

Although pesticides are not typically applied directly to prairie strips, their use is not restricted in the surrounding fields. Pollinators in strips can experience agrochemical exposure through multiple pathways, depending on the application method, crop, time of year, and pest pressure (box 2, figure 1). Drift from foliar applications can directly contact pollinators or contaminate the leaves or flowers of plants in prairie strips downwind of a treated crop field. Neonicotinoid insecticides formulated in seed coatings can dissolve and move with overland runoff and subsurface flow (Hladik et al. 2014). Because of the systemic nature of neonicotinoids, they can then be taken up by nontarget plants (Hladik et al. 2017, Hall et al. 2022). Seed coatings can also be abraded during planting, resulting in contaminated planter dust drifting into surrounding noncrop areas (Krupke et al. 2012). In all cases, off-field movement can result in contaminated pollinator habitat. Even without drift, pollinators that forage within cropland could experience contact exposure or collect contaminated materials and could return pesticides to their nest. Although on-farm research of prairie strips generally shows positive outcomes for wild and managed pollinators (as is summarized in the present article), measuring pesticide exposure and assessing risk are imperative for evaluating the costs and benefits of this practice.

Several studies have assessed the fate and transport of neonicotinoids in prairie strips. When prairie strips were installed at the base of fields planted with treated seed, neonicotinoid levels in the crop field were reduced 2–3 years later (Hladik et al. 2017). In addition, in years 2–3, no neonicotinoid residues were detected in plant foliage or roots within the prairie strips. The fact that residues in plants were below the 1 nanogram per gram limit of detection suggests growth dilution or degradation following initial treated seed planting (Hladik et al. 2017). A subsequent study of a watershed with ongoing seed treatment use found prairie strips did not eliminate downslope insecticide runoff of the neonicotinoid clothianidin; the sampled forbs in prairie strips had relatively low clothianidin levels—for example, well below the known lethal doses for insects (Rutkoski et al. 2024). In a third study, soil and plant tissue from prairie strips showed high incidence of neonicotinoid detections (albeit at low concentrations) when adjacent to fields planted with neonicotinoid-treated seed-- 100% of the soil samples, 80% of the flowering plant leaf tissue, and 80% of milkweed leaves, the larval host of monarch butterflies (Hall et al. 2022). However, the concentrations reported were similar to prior studies of nonprairie strip watersheds (Berens et al. 2021) and field margins (Hall 2021) in the region. Combined with the results from Hladik and colleagues (2017), these studies suggest neonicotinoids can indeed reach plant material in prairie strips that is contacted by pollinators, but concentrations are low and variable over space and time and are not different from those in the crop-adjacent and noncrop environments in the surrounding landscape.

Additional data more directly addressed pesticide levels in pollen and nectar within prairie strips. In a honey bee study, pesticide levels in forager-collected pollen and nurse bees were similar between apiaries in prairie strips or in crop field margins (Hall 2021). This finding suggests that prairie strips do not increase pesticide exposure to honey bees above that which they are receiving in conventional fields. Furthermore, neonicotinoids and foliar-applied insecticides and fungicides were rarely detected in both nurses and pollen; the median levels were below the limit of detection of 0.5 nanograms per gram (Hall 2021). Pollen from wild bumble bees and managed honey bees caught foraging on flowers in strips had pesticides in 40% of pollen samples and less than 10% of nectar samples, but again, the median levels were below a 0.5 nanograms per gram limit of detection (Hall 2021). However, this and prior studies quantifying pesticide concentrations in prairie strips document variability likely because of application concentrations, seed densities, target plants sampled, and land-use histories.

### Potential adverse effects on pollinators

To fully understand pesticides' impacts on pollinators, exposure levels must also be paired with information on the effects of field-realistic concentrations on different pollinator species. With regard to monarch butterflies, the maximum observed concentrations of neonicotinoids in milkweed leaves (Hall et al., 2022) and forbs in prairie strips (Rutkoski et al. 2024) were well below acute LC_10_ values (concentration at which 10% of tested individuals die) for monarch butterfly larvae, and 10 to 130 times lower than chronic LC_10_ values for monarch larvae (Krishnan et al. 2021). In addition, adults consuming even the highest reported neonicotinoid concentrations in wildflower nectar are predicted to be at minimal mortality risk (Krishnan et al. 2021).

An even more in-depth analysis for honey bees leveraged publicly available pesticide toxicity metrics for both larvae and adults (e.g., LD₅₀, a lethal dose that kills 50% of tested individuals, and where there were no observable adverse effect levels), and risk quotients were calculated by dividing measured environmental concentrations by these toxicological endpoints. For neonicotinoids specifically, 6-week colony feeding studies were also incorporated, providing a more integrated perspective on potential sublethal and chronic effects. For all but one chemical assessed, the level of concern (a value used by the USEPA to determine potential harm to nontarget organisms) was not exceeded, even under worst-case exposure scenarios (Hall 2021). Although the maximum levels of thiamethoxam found exceeded the adult chronic risk quotient (a risk quotients greater than 1.0), over 90% of measured values from prairie strips forbs were under 0.5 nanograms per gram (i.e., the lowest amount that can be quantified using highly sensitive liquid chromatography tandem mass spectrometry instrumentation). Furthermore, when comparing when there were no observable adverse effect levels from colony feeding studies with these maximum concentrations, the exposure levels found in prairie strips were more than seven orders of magnitude lower, indicating minimal risk to honey bees under typical environmental conditions (Hall 2021, O’Neal and Hall 2024).

The overall picture emerging from these studies can be summarized as follows. Neonicotinoids in nontarget plant leaf, pollen, and nectar concentrations in prairie strips are typically below levels of detection and when detected the concentrations are below the USEPA’s levels of concern. When measured concentrations of foliar applied insecticides (organophosphates and pyrethroids) and fungicides (strobins) are detected in prairie strips, likely because of spray drift, current best available data suggests insecticide concentrations are below the USEPA’s level of concern for acute and chronic exposures (there are currently no published strobin-specific toxicity thresholds). Finally, pesticide exposure experienced by pollinators may be variable in time and space, but the current evidence based on monitored concentrations and published toxicity thresholds (based on model insects such as honey bees and monarchs) suggests a low likelihood of mortality.

Although assessing risk using typical toxicity endpoint data is important, a large body of evidence using honey bees as a model or surrogate species shows sublethal pesticide exposure can negatively affect pollinator physiology and behavior (Siviter et al. 2021, Tosi et al. 2022). Pesticides can also interact with other environmental factors to produce additive or synergistic effects. Improved diet quality can reduce the toxicity of multiple classes of pesticides (Barascou et al. 2021) and increase tolerance to viral infection (Dolezal and Toth 2018). Using pesticide exposure data from prairie strips, Hsieh and Dolezal (2024) tested how pesticides, nutrition, and pathogen infection interact in a laboratory setting. As was expected from the risk calculations (Hall 2021), bees fed chronic doses of pesticides representing worst case scenario concentrations from the aforementioned exposure studies resulted in no toxicity in honey bee workers, regardless of which diet they consumed. However, when a third stressor, pathogen infection, was introduced, the patterns became more complex and context dependent. When an artificial diet was consumed, pesticide exposure increased virus-mediated mortality (i.e., sublethal pesticide contamination made infections worse); however, when bees consumed natural pollen, some pesticides actually improved the bees' responses to infection, likely through a hormetic mechanism. This response was not identical for all insecticides tested, showing complex context dependency in predicting how pesticides, nutrition, and pathogens interact (Hsieh and Dolezal 2024). Although generated in a somewhat artificial laboratory setting, these data support a growing literature demonstrating that pesticide effects on pollinator health can interact in complex ways with other environmental stressors. Overall, Hsieh and Dolezal (2024) provides experimental evidence that high-quality diets (such as those found in prairie strips) can buffer bees from levels of insecticides typical in prairie strips. However, when combined with multiple stressors (such as virus infection), worst case scenario pesticide exposure from strips has some potential for negative health impacts. Nonetheless, when taken together with the results from field studies (reviewed above), honey bees can experience net benefits from prairie strips, even in the presence of some level of pesticide exposure.

Extrapolating the full impact of pesticides on pollinators also requires an improved understanding of the risks unique to wild bees (Raine and Rundlöf 2024). There is substantial variation among wild pollinators in life history traits such as sociality, diet (e.g., whether they consume plant leaf tissue, or are specialist foragers), and habitat requirements (Knapp et al. 2023). Unlike honey bees, many wild bees and wasps nest in the soil (Harmon-Threatt 2020), and ground nesting species can be exposed to pesticides, especially neonicotinoids, through consumption of soil water or cuticular contact to contaminated material in the cavity (Kopit and Pitts-Singer 2018, Raine and Rundlöf 2024). Soil contamination with neonicotinoids can result in nontarget exposure during bee development, which could reduce health and survivorship later in life (Anderson and Harmon-Threatt 2019, Raine and Rundlöf 2024). Cavity-nesting bees can also be exposed to pesticides (Kopit and Pitts-Singer 2018), and sublethal exposure can reduce their reproductive output over multiple generations (Stuligross and Williams 2021). Although they are less studied than in honey bees, there is some evidence that floral resources and high-quality diets (i.e., nutritious pollen) can buffer the effects of insecticides in some wild bee species (Costa et al. 2022, Rundlöf et al. 2022). However, to what extent the low concentrations of neonicotinoids and other pesticides found in the soil (Hall et al. 2022, Rutkoski et al. 2024) of prairie strips can have on these pollinators has not been fully evaluated.

There is a lack of detailed toxicity or exposure studies specific to wild bee pollinators related to prairie strips, a knowledge gap that is also true for most agroecosystems and pollinator species. Studies of pollinator communities, although they are not a substitute for this lack of knowledge, are frequently used to provide insights into potential costs and benefits of pollinator habitat creation. More abundant and diverse pollinator communities that persist over multiple years can be used as indicators of positive population outcomes. In prairie strips, overall wild bee communities are more abundant and diverse (Kordbacheh et al. 2020) alongside more robust plant–bee networks in prairie strips than in control field margins (Borchardt et al. 2023), with some bee species showing improved body condition indicators (Borchardt et al. 2023). Although these data suggest forage or habitat benefits can outweigh pesticide risks, landscape-scale risk assessments for more species of flower-visiting insects are needed to better evaluate the potential for pesticide applications to affect pollinator health and create population sinks (Grant et al. 2021).

## Synthesis and future directions

Below, we consider the totality of available evidence to assess what is currently known about net effects (considering both benefits and costs) of prairie strips on pollinators, while acknowledging several areas in need of further study.

### Prairie strips are beneficial to pollinators

In summary, farms with prairie strips can provide forage benefits to pollinators and support more diverse and abundant pollinator communities than farms without prairie strips, even with conventional use of pesticides. In addition, because prairie strips consist of perennial vegetation within soil that remains untilled, they provide habitat for soil-dwelling and ground-nesting pollinators. Although pesticides are present in prairie strips, these exposures are generally at low levels, well below the concentrations expected to cause adverse effects in well-studied taxa such as honey bees and monarch butterflies. Importantly, the pesticide levels in prairie strips do not differ from those in the noncrop plants in the surrounding landscapes. Therefore, the weight of evidence suggests that prairie strips cannot be an ecological trap because they are unlikely to be drawing bees away from nearby higher-quality habitat (box 3, figure 2). Higher-quality habitat for pollinators rarely exists in these agriculturally dominated landscapes. Instead, there is ample evidence that prairie strips provide forage and nutritional benefits to both wild pollinators and managed bees in landscapes that are otherwise depauperate. In addition, the experimental evidence reviewed above in honey bees suggests that high-quality food sources make them more likely to withstand pesticide exposure, including the highest (worst case scenario) levels of pesticide exposure found in prairie strips. Increased abundance of wild bees and monarch butterflies and diversity of bee communities, along with more complex plant–pollinator networks predicted to better support rare species, are clear benefits of prairie strips. Monarch exposure to neonicotinoid insecticides is at concentrations below the levels that elicit adverse population responses (Grant et al. 2021, Krishnan et al. 2021). Although toxicity tests on many wild pollinator species are lacking, limiting our current ability to extrapolate on pesticide costs, there are clear positive effects such as forage and nesting habitat in prairie strips, which have the potential to outweigh pesticide costs.

As was reviewed above, there are clear benefits of prairie strips for honey bee apiaries. In the Midwestern United States and other regions of the world, beekeepers have become accustomed to extremely high levels of colony loss and economic uncertainty (Bruckner et al. 2023). Prairie strips can provide tangible benefits to beekeepers in such landscapes; moving bees to high-quality forage habitat in the late season, such as prairie strips, may be cheaper and less time intensive than feeding the bees. Although pesticide exposure is possible in prairie strips, the likelihood of sublethal adverse effects is modulated by improved nutrition, which suggests minimal health impacts. The risks of adverse effects could be further reduced with the adoption of low-drift application practices and the judicious use of pesticides as part of integrated pest management strategies. Bolstered by support from government incentives through the Conservation Reserve Program, enrollment in CP-43 prairie strips continues to rise (Giese et al. 2024). If this trend continues, the potential to improve the quality of habitat for wild pollinators and for honey bee health and honey production is tremendous. The benefits of prairie strips are even stronger when additional benefits beyond pollinators are considered (figure 3). Furthermore, prairie strips are well aligned with the human dimensions of private lands conservation (box 5). The overall benefits they provide span environmental, cultural, and economic realms and include those that appeal to farmers, farmland owners, and broader society (figure 3). For these reasons, they are uniquely poised to become important contributors to pollinator conservation.

**Figure 3.**
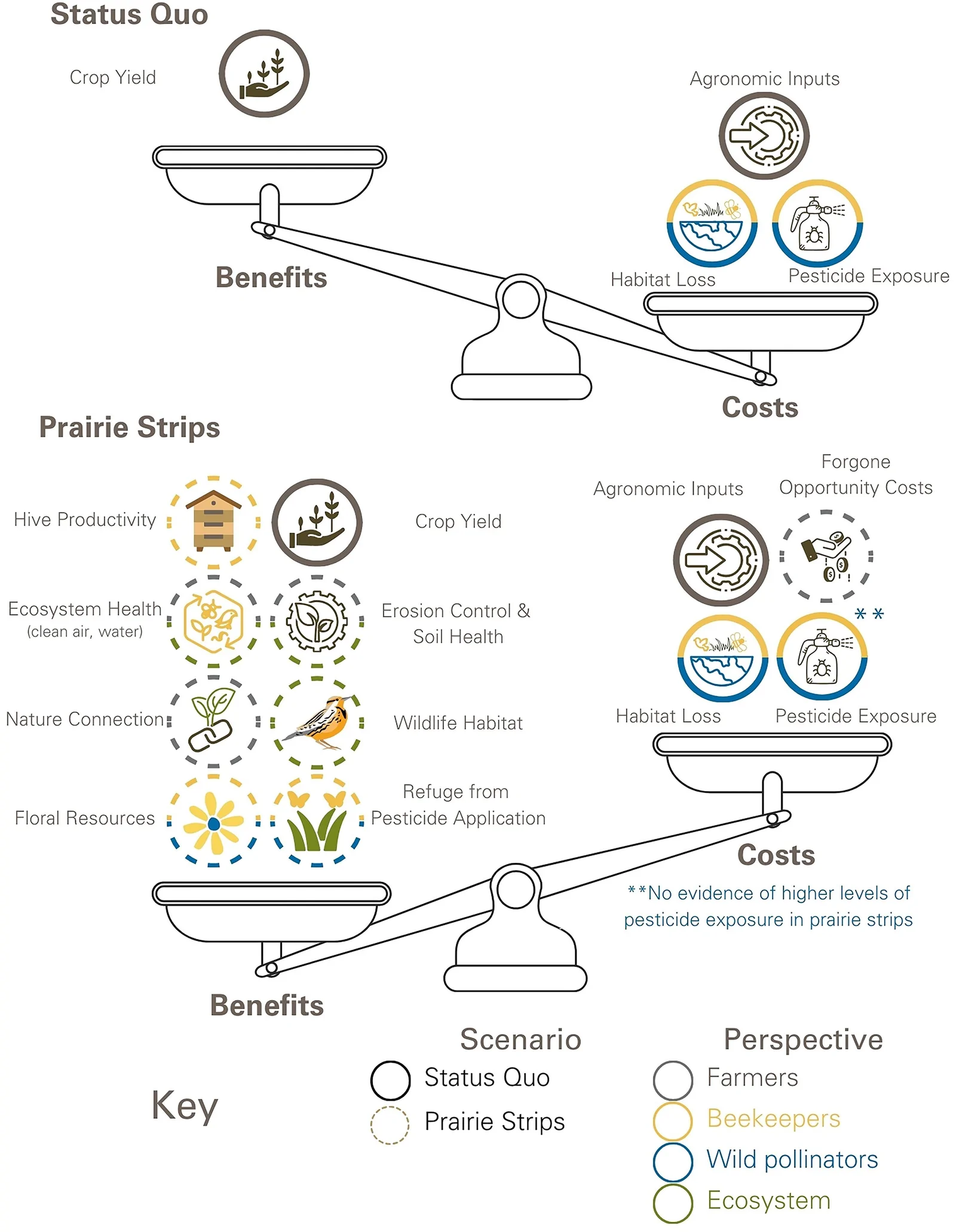
Tipping the balance. Two scenarios for systems-level cost–benefit relationships, considering pollinators within the human dimensions of farming, environmental health, and beekeeping. The perspective (indicated by different color circular outlines described in the key) refers to who is experiencing the costs or benefits. The scenario (indicated by solid or dashed circular outlines) refers to whether the factor is being considered for farms with or without prairie strips. Top: With the status quo, intensive corn–soybean agriculture without prairie strips, the costs outweigh the benefits. Bottom: With prairie strips embedded within the status quo corn–soybean agricultural system, the balance shifts, and the benefits far outweigh the costs. Because the relative weights of costs and benefits are measured in different units and in some cases are unknown, all factors are, by default, assigned circles of the same size.

Box 5. **The human dimensions of conservation adoption.**

Taxpayer-funded federal programs (such as CP-43, prairie strips) involve multiple stakeholders, including farmland owners, farmers, beekeepers, and the broader public. Farmland owners implement and are responsible for the initial and long-term costs of prairie strips, but these costs (e.g., for establishment, management, and land) can be shifted in part or whole to the public through participation in government conservation land-rental and cost-share programs. Landowners who implement prairie strips have diverse attitudes, beliefs, and behaviors about conservation (Upadhaya et al. 2021). Their motivations to adopt and maintain such practices are complex, variously involving agronomic, financial, personal, sociological, and institutional factors (Luther et al. 2020). Prairie strips have advantages over alternative practices, including comparatively low initial and management costs and high cost-effectiveness (Tyndall et al. 2013). They also offer the potential for jointly producing environmental and production benefits, an outcome significantly appealing to landowners and farmers (Wossink and Swinton 2007, Luther et al. 2020). Farmland with prairie strips may be rented, and in this case, prairie strip use and management would also involve a tenant farmer in addition to the farmland owner.

There are no currently known benefits of honey and wild bee pollination to soybean yield in the US Midwest, where prairie strips are implemented. Therefore, crop pollination is not currently an incentive for farmer adoption. Prairie strips may, however, have a positive impact on personal use or revenue-generating recreation for hunting, birding, beekeeping, and agrotourism (Wicks and Merrett 2003). Many farmers with prairie strips may be primarily interested in long-term agronomic benefits through soil conservation, some also report return of wildlife and biophilia as a benefit (Schulte Moore 2026). Recent surveys suggest many Iowa landowners and farmers who produce row crops are distinctly interested in helping pollinators (Cass et al. 2022). Additionally, many landowners are willing to have apiaries on their land in support of local honey production (Cass et al. 2022). Although there is currently strong evidence that prairie strips will not harm honey bees and other pollinators overall, Cass and colleagues (2022) suggested that beekeepers overestimate the negative impacts of pesticides and undervalue the importance of forage nutrition, so it is possible that the role of prairie strips to this industry may be undervalued.

The public broadly supports agricultural conservation measures, and their perspectives shape conservation programs. Khanal and colleagues (2022) found that Iowa residents are willing to pay for expanded use of prairie strips and value increased pollinator habitat as the second most valuable attribute of the practice, behind nutrient retention for water quality. Coordination with and between landowners, along with national and local programs to incentivize region-wide efforts, will be essential to create enough habitat at a landscape scale to reverse pollinator declines in agroecosystems (Salliou et al. 2019).

Because agricultural land is predominantly privately owned and actively farmed, opportunities to improve habitat for pollinators in industrialized agricultural landscapes are limited. Employing prairie strips in and around monocultural row-crop production can provide an opportunity to support pollinator health that is not attainable in grass dominated conservation practices, which experience pesticide exposures similar to those reported in strips but without the benefits of nectar sources. For example, if a pollinator visits a flower in a terrace, grassed waterway or a grass filter strip, they would face a pesticide exposure level similiar to that of a flower in a prairie strip but without the additional floral diversity and nutritional benefits. Wider adoption of cover crops would be a boon for soil and water conservation, but the most frequent cover crop used in this region is the nonflowering grass cereal rye (*Secale cereale*), which also does not provide additional floral diversity or nutrition. If a flowering cover crop (e.g., hairy vetch, *Vicia villosa*) is planted, it is often removed with an herbicide application before all the flowers are fully developed (Clark 2007). This application can also be accompanied by an extra application of insecticides. Therefore, prairie strips currently represent one of the highest-quality pollinator habitat options available for this region.

### Knowledge gaps

Although prairie strips have been thoroughly researched for over 15 years, resulting in a wealth of information about this system’s potential for agronomic (Dutter et al. 2023), environmental (Schulte et al. 2017), and pollinator benefits (reviewed in the present article), there are inevitably gaps in our knowledge. First and foremost, the weights of the relative costs and benefits of each factor considered in the present article (figure 3) remain difficult to assess. These will depend on which perspective is taken (ecological, agronomic) and which metrics of costs and benefits are used (monetary, biodiversity, ecosystem services). In addition, there are important considerations of scale; for example, does the surrounding landscape (e.g., availability of other pollinator habitat) or use of other pollinator-friendly practices in a region affect the practice’s efficacy to support pollinators? As such, there are many areas for future research that can help better understand the full potential of prairie strips to support pollinator health and conservation.

One of the most significant current knowledge gaps in pesticide research is understanding their impacts on a broader range of pollinator species, particularly beyond honey bees (*Apis mellifera*) and monarch butterflies (*Danaus plexippus*). Some studies suggest that, at the individual level, extrapolating from *Apis* in both acute contact and oral toxicity scenarios is unlikely to underestimate non-*Apis* bee toxicity (Thompson 2015, Siviter et al. 2021). However, it is still uncertain whether *Apis* represents a good surrogate species at the colony or population levels. Non-*Apis* bees often encounter distinct exposure pathways and show variable sensitivities because of differences in their biology, behavior, and ecology factors not adequately captured by honey-bee-based assessments. In addition, we lack information on how pesticides in prairie strips soil will affect pollinators, along with basic information about the locations and densities of ground nesting species. This is a major knowledge gap, because at least 70% bee species are ground nesting (Christmann 2022), and soil-based exposure has been demonstrated to have negative effects on the populations of some wild bees (Willis Chan and Raine 2021). As Raine and Rundlöf (2024) emphasized, species-specific data and more inclusive risk assessment frameworks are urgently needed to ensure the protection of diverse pollinator communities.

We also lack information about prairie strips as buffers against pesticide drift or residue accumulation. Although one study suggested the potential for prairie strips to reduce some soil-borne pesticides after 2–3 years (Hladik et al. 2017), much remains unknown about the effects of levels of toxicity on pollinators and the long-term effects. In addition, because they are not directly sprayed, prairie strips have the potential to buffer insects from insecticides, but it is not known to what extent they provide a refuge from such sprayed insecticides. If prairie strips are demonstrated to significantly mitigate pesticides in soil, plants, or air, this would add to their potential benefits to pollinators. Although it is not yet known whether herbicide drift affects plant abundance, diversity, floral resource density, and nutritional quality in prairie strips, the Iowa-based studies reviewed in the present article occurred in herbicide-treated fields, showing the potential for pollinators to benefit even despite herbicide use. In addition, it will be important to address the potential impacts of commonly used fungicides used in crops adjacent to prairie strips; these agrochemicals can affect bee health (Schuhmann et al. 2022) and can potentially indirectly affect bees via alterations in soil microbial communities (Diaz et al. 2023). Pest control strategies change over time in response to pressures from changing pests, policies, economics, and technologies. Therefore, it will be important to continuously assess and reassess pesticide effects on pollinators in the context of prairie strips to make sound recommendations to practitioners aiming to provide maximal benefits to pollinators.

Prior research on prairie strips, as well as in Midwestern farmland and restored prairie systems, suggests there is high seasonal variation in floral resources, with the late summer emerging as a particularly challenging time for pollinators (Dolezal et al. 2019). Because prairies are rich in late-blooming forbs, they have great potential to provide floral resources over a longer period of time (Zhang et al. 2023), and this may partially explain why they support more diverse pollinator communities (Kordbacheh et al. 2020). In the future, research examining different seed mixes and considering plant species’ phenologies could provide insights into further improvements in prairie strips as pollinator habitat. Besides floral resources, prairie strips also have great potential as pollinator nesting habitat; future research should aim to elucidate how different soil characteristics, nesting site availability, and potential interactions with tillage practices can be considered to best support ground-nesting species. Prior research suggests prairie strips may also increase the abundance of some natural enemies of crop pests (Cox et al. 2014, Vargas 2024); future studies can address whether there are additional agronomic benefits of prairie strips through increased pest control in adjacent crops. Another promising area for future work is the use of prairie strips in the context of other cropping systems that are dependent on insect pollination; if pollinators benefit alongside these crops, farmers have the potential to realize increased crop pollination as an additional agronomic benefit of prairie strips (Garibaldi et al. 2021b).

### Lessons beyond prairie strips

Although we focus on prairie strips in the Corn Belt of the United States, there are other types of pollinator habitat enhancements being implemented globally, including on farmland, in urban parks and yards, and along roadsides (Gill et al. 2016). Prairie strips can serve as a model for ecological cost–benefit analysis and are therefore relevant to other types of landscape enhancements for pollinators such as hedgerows, pollinator gardens, solar installations with pollinator habitat. The message from prairie strips research is promising and suggests that the benefits of improved floral resources have the potential to outweigh the costs of pesticide exposure (figure 2). This is especially good news, given the fact that the amount of land being placed in prairie strips on a single farm is relatively small (usually less than 4 hectares or less than 10% of a crop field), but even small plantings of diverse, native species implemented by individual farmers can have positive impacts. These findings are in line with other recent research in the Upper Midwestern United States, suggesting the presence of a diverse native plant community can trump the effects of neonicotinoid exposure on pollinator visitation and arthropod abundance (Tetlie, 2025), supporting the notion that high-quality reconstructed habitat can provide numerous benefits that outweigh insecticide impacts.

As a pollinator conservation practice, we do not recommend prairie strips as a replacement for the larger-scale habitat restoration efforts that may be required to meet the conservation needs of specific species. Conversely, the need for large-scale conservation projects should not prevent efforts such as prairie strips. Prairie strips are a more realistic solution than reserving large tracts of especially expensive, high-yielding agricultural land for biodiversity (Garibaldi et al. 2021a) and are accompanied with several cobenefits (Schulte et al. 2017). In addition, the use of pesticides in the surrounding landscape should not be viewed as a deterrent to the establishment of practices such as prairie strips, given the potential for net benefits. At the same time, reductions in the use of pesticides in agroecosystems and the increased use of low-drift application technology can further enhance the value of practices such as prairie strips as pollinator habitat. Prairie strips established in landscapes with low pesticide use are more likely realize maximal benefits as pollinator habitat. Recent research suggests many prophylactic applications of insecticides are unnecessary or only marginally effective and are not worth the risk to beneficial insects such as pollinators (Pecenka et al. 2021). Therefore, integrated pest management practices that reduce agrochemical use, hand in hand with practices such as prairie strips, are a promising way forward to maintain agronomic bottom lines while also protecting pollinators (Leach et al. 2022).

In the European Union, practices such as flowering strips and hedgerows are also supported through governmental programs such as the European Union's agri-environment schemes. A meta-analysis indicated that such practices generally have positive influences on pollination services, but this depended on the age of the planting, the plant's composition, and the surrounding landscape (Albrecht et al. 2020). Positive pollinator responses were strongest in simple landscapes (cropland dominated), and more diverse plantings were more effective at supporting pollinators (Scheper et al. 2013). However, these effects may vary with the cropping system and with its level of pollinator dependence. In some cases, mass-flowering crops can have positive effects on some wild bee species (Holzschuh et al. 2013); therefore, the relationships between agricultural intensification, landscape context, and pollinator responses can be complex. Nonetheless, combined with our results on prairie strips, there is mounting evidence that habitat enhancements and pollinator-friendly practices within farms have the potential for net positive effects across a wide variety of crops, landscapes, and global regions.

Prairie strips may not be a good model for all systems, however, and these results must not be blindly extrapolated without system-specific data on pesticides, forage, bee health, and pollinator communities. For example, in areas with greater amounts of natural or seminatural land, such as around public parks, forests, and wildlife refuges, these dynamics may be different. The findings from prairie strips are more likely to be applicable to landscapes dominated by human activity. In locations where more than 75% of the landscape is in agriculture with widespread pesticide use, the amount of habitat free from pesticide exposure is likely to be low to nonexistent (Meehan et al. 2011).

### Conclusions

The global loss of biodiversity including pollinators because of agriculture is one of several pressing environmental challenges of our time. Although large-scale reserves are needed to achieve conservation goals, establishing them in areas already in agricultural and urban land uses is challenging because of the high opportunity costs of removing land from more economically lucrative uses. In the present article, we synthesized a decade of research revealing an improvement to habitat for pollinators with the establishment of prairie strips, a land-sharing strategy gaining widespread traction in the highly agricultural US Corn Belt. We conclude that, despite occasional exposure to pesticides, the balance of the evidence to date shows that prairie strips help expand and improve pollinator habitat in these otherwise depleted landscapes. We recommend further research to understand how establishing small patches of flowering native plants effects pollinators in other regions. We also recommend continued promotion of integrated pest management strategies within agricultural communities to reduce pesticide risk to pollinators and maximize net benefits of pollinator habitat in farmed landscapes.

## Funding

This work and several cited studies by the authors were supported by the following grants: United States Department of Agriculture (USDA)-National Institute of Food and Agriculture (NIFA) grant no. 2017–68004-26326 to ALT, AGD, and MEO; USDA-FSA grants no. AG-3151-C-0041, no. AG-3151-P-14–0065, and no. AG-3151-P-14–0162 to LASM; USDA-NIFA grant no. 2015–67019-23002 to LASM; Iowa Soybean Association grant no. Y8CWNJRCNN91 to AGD, MEO, LASM, and ALT; and grant no. 549025 from the Foundation for Food and Agricultural Research to LASM, ALT, AGD, MEO, and JCT. The aforementioned FFAR grant involved matching funds from Bayer Crop Science, DuPont Pioneer (now Corteva Agriscience), the College of Agriculture and Life Science at Iowa State University and the Graduate Program in Sustainable Agriculture, and Syngenta; these funders were not involved in determining or executing the scope of work.

## Data Availability

There are no new data associated with this article.
